# Impact of social exclusion on empathy in women with borderline personality disorder

**DOI:** 10.1007/s00406-022-01535-0

**Published:** 2023-01-05

**Authors:** Livia Graumann, An Bin Cho, Eugenia Kulakova, Christian Eric Deuter, Oliver T. Wolf, Stefan Roepke, Julian Hellmann-Regen, Christian Otte, Katja Wingenfeld

**Affiliations:** 1 Charité-Universitätsmedizin Berlin, corporate member of Freie Universität Berlin and Humboldt-Universität zu Berlin, Department of Psychiatry and Neurosciences, Campus Benjamin Franklin, Berlin, Germany; 2 Department of Cognitive Psychology, Institute of Cognitive Neuroscience, Ruhr University Bochum, Bochum, Germany

**Keywords:** Borderline personality disorder, Social cognition, Empathy, Cyberball, Social exclusion

## Abstract

Unstable interpersonal relationships and fear of abandonment are core symptoms of borderline personality disorder (BPD) that often intensify during stress. Psychosocial stress, which includes components of social exclusion and increases cortisol secretion, enhances emotional empathy in healthy individuals. Women with BPD, on the contrary, react with reduced emotional empathy. The aim of the present study was to investigate the effects of perceived social exclusion without accompanying cortisol increase on empathy in women with BPD and healthy women. To induce social exclusion, we randomized 98 women with BPD and 98 healthy women to either an exclusion or an overinclusion (control) condition of Cyberball, a virtual ball game. Subsequently, participants underwent the Multifaceted Empathy Test (MET), which assesses cognitive and emotional empathy. There was no increase in cortisol release after Cyberball. Cognitive empathy did not differ between groups or conditions. Women with BPD reported lower emotional empathy for positive emotions (group by valence interaction), but not for negative emotions. Exploratory analyses suggested that this effect might be more pronounced after social exclusion. Our results confirm previous findings that cognitive empathy does not differ between women with BPD and healthy women and extend this evidence to social exclusion. Emotional empathy in women with BPD seems to be more sensitive to the effects of stress or ambiguous social situations. Specifically, emotional empathy seems to be reduced for positive emotions, and might further decline after social exclusion. Empathic reactions to emotional stimuli of different valences and to specific emotions should be further investigated.

**Supplementary Information:**

The online version contains supplementary material available at 10.1007/s00406-022-01535-0.

## Introduction

Unstable interpersonal relationships and fear of abandonment are among the defining and most debilitating symptoms of borderline personality disorder (BPD; [1, 24]). Results of several studies suggest that social cognition in patients with BPD is impaired: patients with BPD are more sensitive to social rejection and negative evaluation and show broad expectancies of rejection across situations [7, 8, 35]. An important prerequisite for adequate social interaction is empathy, which comprises at least two components. Cognitive empathy is the ability to infer others’ mental states, which is closely related to the concept of theory of mind, and also referred to as mentalizing. Emotional empathy can be regarded as the ability to emotionally respond to another person’s emotional state [10, 57].

Many of the disorder’s core symptoms typically occur in interpersonal contexts [36] and worsen under perceived stress [27, 32]. Acute stress activates the hypothalamic–pituitary–adrenal (HPA) axis, which initiates a range of adaptive hormonal and behavioral responses including release of the glucocorticoid hormone cortisol [46, 56]. In addition, stress activates the sympathetic nervous system (SNS), which is frequently measured using salivary alpha-amylase (sAA) as a marker [30]. There is growing evidence that HPA-axis function in patients with BPD is altered and that cortisol reactivity to psychosocial stress is attenuated (for a meta-analysis, see [15]). In one of our previous studies, we investigated empathy under psychosocial stress in women with BPD [52]. Stress as elicited with the Trier Social Stress Test (TSST; [31]), resulted in lower emotional empathy in women with BPD, but elevated emotional empathy in healthy women. Cognitive empathy remained unaltered.

In line with results of other studies showing enhanced prosocial behavior [12, 45, 47] and heightened emotional empathy [55] after stress, these results suggest a prosocial reaction to psychosocial stress in healthy individuals. These findings support the “tend-and-befriend” hypothesis, stating that (psychosocial) stress leads to increased prosocial behavior [5, 44], instead of a “fight-or-flight” response to stress [6]. Within a social context, tend-and-befriend behavior seems to be a functional coping mechanism to reconnect with others [33].

In another study, pharmacological stimulation of the mineralocorticoid receptor (MR), one of the cortisol receptors in the brain, resulted in increased emotional empathy in healthy controls (HC) but also in patients with BPD [53]. Results suggest that women with BPD are generally capable of emotional empathy and that cortisol release in a non-social context might increase empathy. In patients with BPD, however, psychosocial stress might result in a switch from prosocial tend-and befriend to less adaptive fight-or-flight behavior (i.e., aggression or withdrawal). This does not seem to be attributable to cortisol release alone, but rather to psychological mechanisms such as social exclusion. Indeed, patients with BPD are sensitive to social rejection and negative evaluation and rate the TSST as more threatening compared to healthy controls [52]. The TSST includes the presence of two judges, who are non-responsive to the participant, which might be perceived as social exclusion. We believe that this perception of social exclusion might hinder adaptive coping and result in reduced prosocial behavior and cognition.

An experimental task frequently used to study social exclusion is the Cyberball paradigm [49], a virtual ball-tossing game. It usually consists of an inclusion and an exclusion condition. In the inclusion condition, all players receive the ball to an equal amount. In the exclusion condition, after a first round of inclusion, the participant does not receive the ball any longer and is excluded from the game. Previous studies using Cyberball support the picture of a biased perception of social participation in patients with BPD. Even in inclusion conditions, they reported to receive the ball less often and felt more ignored than controls [37, 41, 42].

While one of the TSST’s main purposes is to induce a cortisol increase, most studies have shown no cortisol increase after Cyberball (e.g., [20, 21, 40]) and no differences have been reported between HC and patients with BPD [28, 29]. Only few studies have investigated sAA responses to Cyberball. While Bass et al. [2] found no Cyberball effects on sAA, Helpman et al. [26] reported higher sAA levels after Cyberball exclusion, but did not include a control condition. In both studies, sAA levels increased over time.

The aim of the present study was to investigate why patients with BPD differ in their emotional empathy response to psychosocial stress from healthy controls. In our model, we assume that the inhibition of prosocial behavior seen in patients with BPD is related to a feeling of social exclusion. Therefore, we used Cyberball to induce social exclusion without confounding of a cortisol increase. Subsequently, participants underwent the Multifaceted Empathy Test (MET) as a measure of cognitive and emotional empathy.

We expected no changes in cortisol or alpha-amylase release after the Cyberball game in women with BPD and healthy women. We hypothesized that women with BPD and HC would not differ in cognitive and emotional empathy in the control condition (overinclusion). In contrast, we expected that social exclusion would result in enhanced emotional empathy in HC and reduced emotional empathy in patients with BPD.

## Method

### Participants

The sample consisted of 98 women with BPD and 98 healthy women, who were matched for age, education, intake of hormonal contraception and menstrual cycle. Women between the ages of 18 and 50 with a BMI between 17.5 and 30 were included. All participants underwent the Structural Clinical Interview for DSM-5 Disorders (SCID) (German versions of SCID-5-CV, SCID-5-PD; [3]). Exclusion criteria for all participants were neurodegenerative, metabolic, endocrine, autoimmune and CNS diseases, any other severe somatic diseases, intake of glucocorticoids and pregnancy. Patients with BPD were excluded if they suffered from acute major depressive episode, psychotic symptoms (lifetime), substance addiction or acute suicidal behavior. To exclude polypharmacy, daily intake of more than three different psychotropic substances or intake of benzodiazepines led to exclusion in patients with BPD. Dosage had to be stable for at least one week at testing. Healthy controls needed to be free of lifetime psychiatric diagnoses, treatment and medication.

We recruited participants via postings on the internet and flyers distributed in the hospital. Additionally, inpatients with BPD were selected at the Department for Psychiatry and Neurosciences, Charité Berlin, Campus Benjamin Franklin, Germany. All participants were informed about the procedure orally and in written form and had to give informed consent before participation. All participants were reimbursed with 60 Euros and were able to earn up to 30 Euros additionally in one of the computer games. The Charité Ethics Committee approved the study.

### Procedure

The study involved two testing sessions. In the first session, diagnostic interviews were conducted by trained clinicians. Subsequently patients filled out several German psychopathology questionnaires on a tablet or a computer in the laboratory, including Beck Depression Inventory (BDI-II; [25]) to assess depressive symptoms and the International Trauma Questionnaire (ITQ; [9]) to assess trauma related symptoms according to ICD-11. Severity of borderline symptoms was assessed with the short version of the Borderline Symptom List (BSL-23; [4]. Participants filled lout the Rejection Sensitivity Questionnaire (RSQ-9, [41, 42]), which assess rejection sensitivity. These data were collected anonymously using the web application RedCap and were processed over a secure web connection with authentication and data logging.

At the second session, participants played Cyberball and subsequently performed the Multifaceted Empathy Test (MET; [19]) and another task, which will be presented elsewhere. Before Cyberball and after MET, participants took the Multidimensional Mood State Questionnaire, a short questionnaire on current mood (MDMQ; [43]). The MDMQ was used to measure changes in mood, wakefulness, and nervousness from before to after the experiment. Additionally, participants filled out the Need Threat Questionnaire (NTQ; [23]), which assesses Cyberball related need threat (e.g., “I felt rejected”) and ostracism intensity (e.g., “I was ignored”). We additionally assessed the degree to which participants believed that they had played the game with real players.

### Social exclusion

Participants were randomized to an exclusion or an overinclusion condition of the Cyberball paradigm [50], a virtual ball game including three players. Because of a biased perception of inclusion in patients with BPD, we used an overinclusion condition as the control condition [11, 48]. Both conditions consisted of 30 ball tosses and ran for two to three minutes. In the exclusion condition, participants received the ball twice within the first six throws, to ensure that they felt included in the game. For the rest of the game, the participant did not receive the ball any longer. In the overinclusion condition, participants received 45% of all throws, i.e., 13 throws.

Before the game, participants read the instructions, stating that they should imagine the game as if it was happening in real life with real people. It also stated that they would be tossing a ball with two real co-players via an internet connection (“cover story”), while in fact these co-players were computer-generated. After completing the whole experiment, all participants were debriefed.

### Empathy

We assessed cognitive and emotional empathy with a modified version of the Multifaceted Empathy Test (MET; [18, 52]). Thirty pictures showing people in different emotional states were presented on a black screen. Seventeen pictures showed people in negative emotional states and 13 pictures in positive states. In alternating order, participants rated blocks of ten pictures for cognitive empathy and then ten pictures for emotional empathy. All blocks were presented twice, once for cognitive and once for emotional empathy.

To assess cognitive empathy, participants were required to infer the mental state of the subject in the photo and to indicate the correct emotion from a list of four. We calculated percentage scores of correct answers. To assess emotional empathy, participants were asked to rate the degree of empathic concern they felt for the person in the picture on a 9-point Likert scale (1 = *not at all*, 9 = *very much*). We calculated mean scores for overall emotional empathy, emotional empathy for negative emotions and emotional empathy for positive emotions [34].

### Physiological outcome variables

Saliva samples for analyses of cortisol and salivary alpha-amylase (sAA) were collected using SaliCap devices (IBL, Hamburg, Germany) at the following time points: baseline (0), after Cyberball (+ 20), after MET (+ 35). Until biochemical analyses, performed in the Neurobiology Laboratory of the Department of Psychiatry and Psychotherapy, Charité, University Medicine Berlin, Campus Benjamin Franklin, Berlin, Germany, samples were stored at − 80 °C. For a detailed description, see supplementary material.

### Statistics

Statistical analyses were performed using SPSS version 27. Demographic variables were analyzed using t-tests for continuous variables and *χ*^2^ tests for categorical variables.

A 2 (overinclusion vs. exclusion) × 2 (BPD vs. HC) ANOVA was carried out for cognitive empathy. Percentage of correct answers served as dependent variable. In a mixed model ANOVA, we evaluated whether emotional empathy differed for negative and positive emotions. Group and Cyberball condition served as between-subjects factors and valence (positive and negative emotions) as within-subjects factor. Mean emotional empathy scores served as dependent variables. Post hoc t-tests were Bonferroni corrected for multiple testing. In addition, we included baseline mood as a covariate into our main analysis on emotional empathy. In the BPD group, we analyzed whether post-traumatic stress disorder (PTSD) diagnosis and intake of psychotropic medication affected empathy in 2 × 2 ANOVAs.

Results on the MDMQ were analyzed using repeated measures (rm-)ANOVAs with condition and group as between-subjects factors and two measurement time points (before and after Cyberball) as within-subjects factors. NTQ scores were analyzed in 2 (overinclusion vs. exclusion) × 2 (BPD vs. HC) ANOVAs as well.

Cortisol and sAA values were log-transformed due to non-normal distribution and were each analyzed using rm-ANOVAs with the within-subjects factor time (0 min, + 20 min, + 35 min) and between-subjects factors group and condition.

## Results

### Demographic and clinical data

Groups did not differ in age, years of school education, use of hormonal contraception, relationship status, phases of menstrual cycle or number of those reporting “no natural cycle”, which include those taking hormonal contraception and menopausal women. There were more smokers and BMI was higher in the BPD group. For results, see Table 1. In the exclusion condition, there were 49 women with BPD and 48 HC. In the overinclusion condition, there were 49 women with BPD and 50 HC. Between conditions, women did not differ in age, years of school education, smoking, phase of menstrual cycle, relationship status or BMI, all *p*s > 0.05. In the exclusion condition, there were more women taking hormonal contraception than in the overinclusion group, (*F*(1, 192) = 156.45, *p* < 0.001, *η*^2^ = 0.45).

**Table 1:** Sample characteristics and psychopathology questionnaire data

Variable	BPD *n* = 98	HC *n* = 98	Statistics
Age (mean/SD)	27.78 (7.23)	27.93 (6.89)	*t*(194) = − 0.15, *p* = 0.880
Years of school education (mean/SD)	11.70 (1.21)	11.96 (1.00)	*t*(194) = − 1.61, *p* = 0.110
Hormonal contraception (y/n)	16/82	16/82	*χ*^2^(1) = 0.00, *p* = 1.00
Smoker (y/n)	42/56	14/84	*χ*^2^(1) = 19.6,* p* < 0.001
Body mass index (mean/SD)	22.84 (3.23)	21.91 (2.46)	*t*(193) = 2.34, *p* = 0.020
Cycle phase (follicular/luteal/no natural cycle)	26/50/20	30/50/18	*χ*^2^(2) = 0.37, *p* = 0.831
In a relationship (y/n)	22/76	29/69	*χ*^2^(1) = 0.13, *p* = 0.254
BDI-II (mean/SD)	27.21 (12.17)	1.74 (2.4)	*t*(193) = 20.31,* p* < 0.001
BSL-23 (mean/SD)	1.96 (.88)	0.08 (.12)	*t*(194) = 20.98, *p* < 0.001
ITQ (mean/SD)	10.57 (6.09)	0.84 (1.64)	*t*(194) = 15.27, *p* < 0.001
RSQ	18.66 (6.54)	6.22 (2.84)	*t*(194) = 17.26, *p* < 0.001

*BPD* borderline personality disorder, *HC* healthy controls, *n* sample size, *SD* standard deviation, *y* yes, *n* no, *BDI-II* Beck Depression Inventory, *BSL-23* Borderline Symptom List-short version, *ITQ* International Trauma Questionnaire, *RSQ* Rejection Sensitivity Questionnaire

Of the BPD group, 47 women were inpatients and 51 were outpatients. Overall, 50 women with BPD reported intake of psychotropic medication and 48 patients with BPD were free of psychotropic medication. The most frequent comorbid diagnosis determined in patients with BPD was PTSD, *n* = 25. For further details on medication and comorbid diagnoses, see supplementary material.

Women with BPD had significantly higher scores than HC on all of the self-report questionnaires for clinical symptoms and rejection sensitivity, all *p* < 0.001. For results, see Table 1.

### Cyberball—manipulation check

Results on the Need Threat Questionnaire (NTQ) indicate that the Cyberball manipulation was successful. Participants reported higher need threat after exclusion than after overinclusion (main effect condition: *F*(1, 192) = 156.45, *p* < 0.001, *η*^2^ = 0.45). Additionally, women with BPD reported higher need threat than HC (main effect group: *F*(1, 192) = 86.52, *p* < 0.001, *η*^2^ = 0.31). There was also a group × condition interaction effect (*F*(1, 192) = 5.83, *p* = 0.017, *η*^2^ = 0.03): women with BPD reacted especially to the exclusion condition with need threat.

**Fig. 1:**
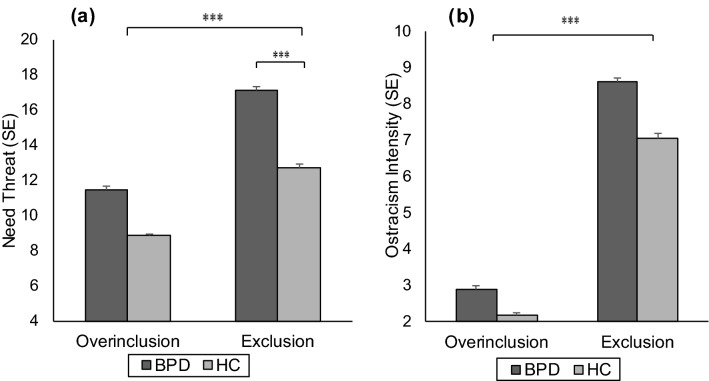
Overall need threat (**a**) and ostracism intensity (**b**) after Cyberball overinclusion or exclusion in patients with borderline personality disorder (BPD) and healthy controls (HC). There were significant main effects of condition and group in need threat (**a**), *p* < 0.001 (***) and ostracism intensity (**b**), *p* < 0.001 (***) and a significant group × condition interaction in **a** need threat, *p* < 0.001 (***). Error bars represent standard error of mean

Participants reported greater ostracism intensity after exclusion (main effect condition: *F*(1, 192) = 496.96, *p* < 0.001, *η*^2^ = 0.72). Additionally, women with BPD reported greater ostracism intensity than HC (main effect group: *F*(1, 192) = 22.37, *p* < 0.001, *η*^2^ = 0.10). Results are shown in Fig. 1.

Women in the overinclusion condition believed that they played with real co-players more than those in the exclusion condition (main effect condition: *F*(1, 189) = 16.47, *p* < 0.001, *η*^2^ = 0.08). Additionally, women with BPD believed the cover story more than HC (main effect group: *F*(1, 189) = 6.33, *p* = 0.013, *η*^2^ = 0.03). Mean values on the NTQ are reported in Table A1 in the supplementary material.

On the Multidimensional Mood State Questionnaire (MDMQ), participants in the exclusion condition reported being more tired and feeling less relaxed than those in the overinclusion condition, both *p*s < 0.05. Women with BPD reported worse mood, feeling more tired and more nervous than HC, all *p*s < 0.001. More detailed results can be found in the supplementary material.

### Physiological data

#### Cortisol

**Fig. 2:**
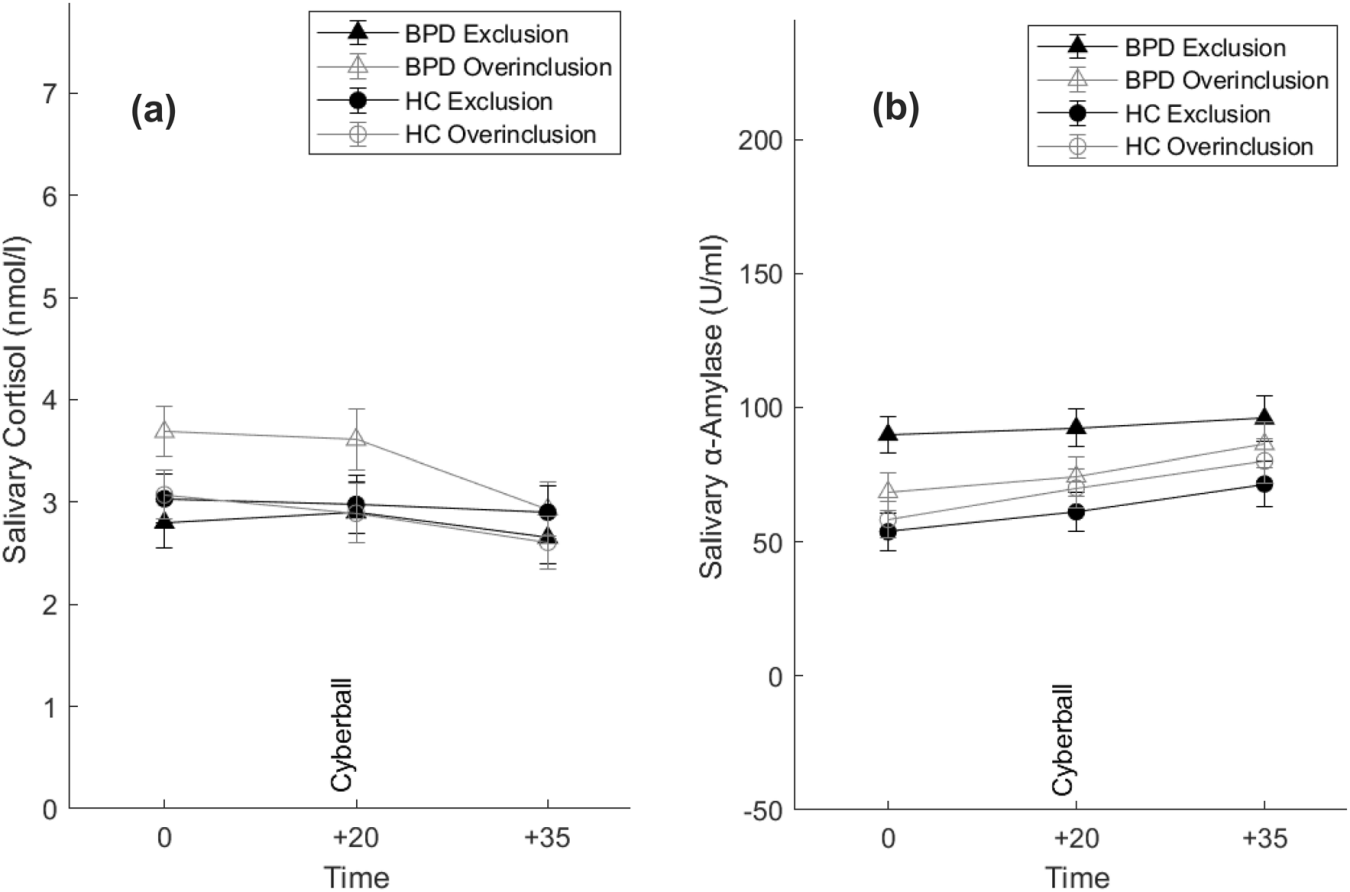
Salivary cortisol (**a**) and alpha-amylase (**b**) concentrations (mean/SE) before and after Cyberball in women with borderline personality disorder (BPD) and healthy women (HC)

We did not find any group, condition or a group × condition interaction effect on cortisol, all *p*s > 0.05. There was a significant main effect of time (*F*(2, 180) = 14.1, *p* < 0.001, *η*^2^ = 0.14), with a decrease in cortisol over time. There were no further significant interactions, all *p*s > 0.05. Results are shown in Fig. 2a.

#### Salivary alpha-amylase (sAA)

We did not find any group, condition or a group × condition interaction effect on sAA, all *p*s > 0.05. We found a main effect of time (*F*(2, 182) = 19.58, *p* < 0.001, *η*^2^ = 0.18) with an increase of sAA over time. There were no further significant interactions, all *p*s > 0.05. Results are shown in Fig. 2b.

### Effects of social exclusion on emotional and cognitive empathy

**Fig. 3:**
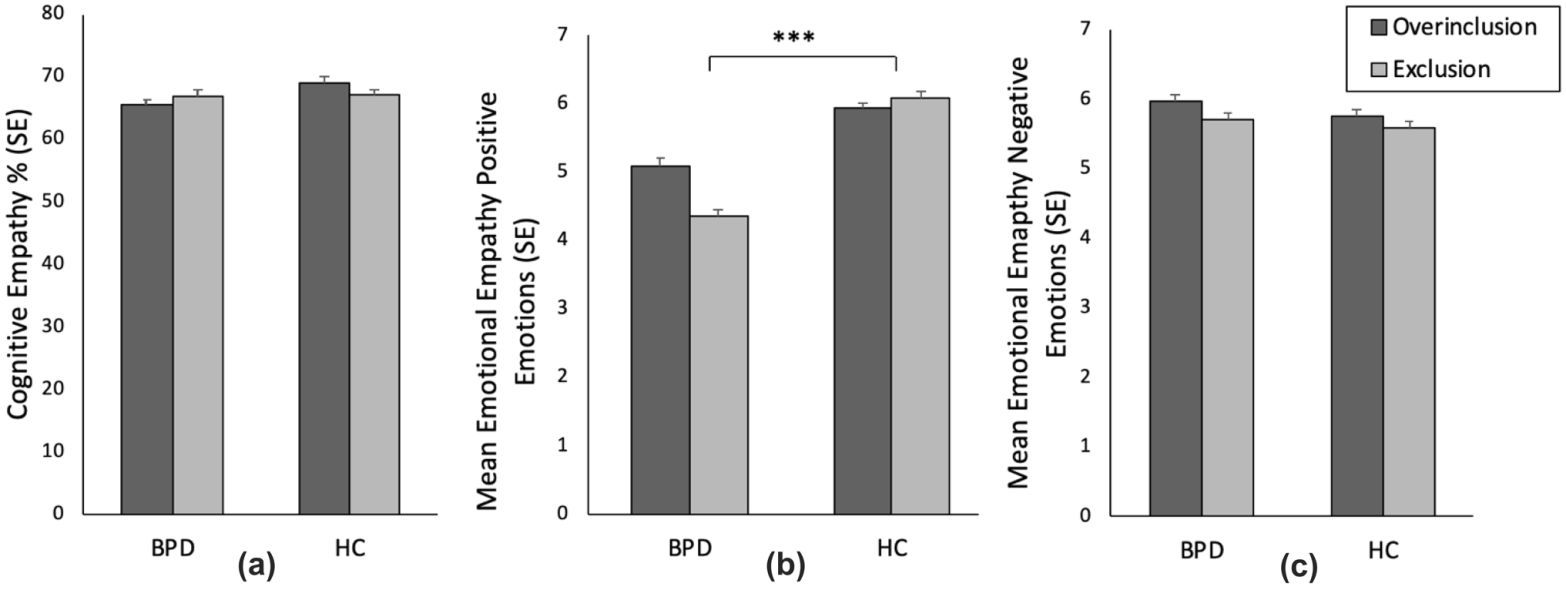
Cognitive empathy (**a**), mean emotional empathy for positive emotions (**b**) and mean emotional empathy for negative emotions (**c**) measured with the multifaceted empathy test (MET) after Cyberball overinclusion or exclusion in patients with borderline personality disorder (BPD) and healthy controls (HC). There was a significant main group effect in emotional empathy for positive emotions (**b**), *p* < 0.001 (***). Error bars represent standard error of mean

Cognitive empathy scores did not significantly differ between the two Cyberball conditions, (*F*(1, 192) = 0.49, *p* = 0.86, *η*^2^ = 0.00) or groups (*F*(1, 192) = 1.0, *p* = 0.318, *η*^2^ = 0.01) and there was no group × condition interaction (*F*(1, 192) = 0.80, *p* = 0.373, *η*^2^ = 0.00). Results are shown in Fig. 3a.

In the analysis of emotional empathy, we found a significant main effect for group, (*F*(1, 192) = 10.01, *p* < 0.001, *η*^2^ = 0.06). Patients with BPD reported lower overall emotional empathy than HC. There was no main effect for condition, (*F*(1, 192) = 2.05, *p* = 0.154, *η*^2^ = 0.01) or a group × condition interaction, (*F*(1, 192) = 1.96, *p* = 0.164, *η*^2^ = 0.01). We found an effect of emotional valence (*F*(1, 192) = 13.00, *p* < 0.001, *η*^2^ = 0.06) and an interaction of group × valence (*F*(1, 192) = 44.66, *p* = 0.002, *η*^2^ = 0.05). Post hoc t-tests revealed that women with BPD exhibited lower empathy for positive emotions than HC (*t*(194) = − 6.10, *p* < 0.001, *d* = − 0.872, 95% CI [− 1.16, − 0.58]). Both groups did not differ in empathy for negative emotions (*t*(194) = 0.80, *p* = 0.424). Results are shown in Fig. 3b and c. Mean values are reported in Table A3.

There was no valence × condition interaction effect (*F*(1, 192) = 0.13, *p* = 0.715, *η*^2^ = 0.00), but a valence × condition × group interaction effect at trend level (*F*(1, 192) = 3.30, *p* = 0.071, *η*^2^ = 0.02). For exploratory purposes, we calculated post hoc t-tests. Women with BPD reported less empathy for positive emotions in the exclusion condition than in the overinclusion condition, (*t*(96) = 2.26, *p* = 0.026, *d* = 0.46, 95% CI [0.06, 0.86]). In healthy controls, empathy for positive emotions did not differ between conditions, (*t*(96) = − 0.59, *p* = 0.560, *d* = − 0.12, 95% CI [− 0.51, 0.28]).

To account for the possible influence of mood on emotional empathy, we included baseline mood on the MDMQ good vs. bad scale as a covariate into our main analysis. We did not find a main effect for group anymore, (*F*(1, 190) = 2.42, *p* = 0.122, *η*^2^ = 0.01). We found an effect of emotional valence (*F*(1, 190) = 14.40, *p* < 0.001, *η*^2^ = 0.07), an interaction of valence × mood (*F*(1, 190) = 10.52, *p* = 0.001, *η*^2^ = 0.05) and an interaction of group × valence (*F*(1, 190) = 4.37, *p* = 0.038, *η*^2^ = 0.02). There was a valence × group × condition interaction effect at trend level (*F*(1, 190) = 3.37, *p* = 0.068, *η*^2^ = 0.02).

We additionally analyzed whether patients with comorbid PTSD differed from those without PTSD. We also compared patients with BPD with and without intake of psychotropic medication. We found no effects of PTSD status or medication intake on cognitive or emotional empathy, all *p*s > 0.05.

## Discussion

We investigated the effects of Cyberball-induced social exclusion on empathy in patients with BPD and healthy controls. Our manipulation was successful, as social exclusion resulted in greater feelings of exclusion compared to the overinclusion condition. Cyberball did not result in an increase in cortisol release. Cognitive empathy did not differ between groups or conditions. Women with BPD reported lower emotional empathy than healthy women in both conditions for positive emotions. Exploratory analyses suggested that this effect might be more pronounced after social exclusion.

### Cognitive empathy

In line with our hypothesis, social exclusion did not change cognitive empathy scores in both groups. This was also found after the TSST [52] and after pharmacological stimulation of the brain’s glucocorticoid [16] and mineralocorticoid receptors [53] in healthy individuals. Similarly, psychosocial stress and mineralocorticoid receptor stimulation did not affect cognitive empathy in patients with BPD [52, 53]. Our findings are also in line with other studies that did not find deficits in social cognition in patients with BPD (e.g., [22, 39]). The present results further strengthen evidence that the ability to correctly identify others’ emotions is not impaired in individuals with BPD and extend this to situations of social exclusion.

### Emotional empathy

Women with BPD showed lower emotional empathy than the control group in response to positive emotional stimuli (e.g., a person showing happiness), but not to negative stimuli. This was also significant after including current mood as a covariate into the analysis. In previous studies, our group did not find reduced emotional empathy in women with BPD in the control conditions, but valence was not taken into account [52, 53]. Domes et al. [14] examined such valence-dependent changes in empathy after administration of oxytocin using the MET. Compared to healthy women, women with BPD exhibited lower emotional empathy for positive emotions, but not for negative emotions in the placebo condition. This difference diminished after receiving oxytocin, which led to enhanced emotional empathy in the BPD group. The present results are in line with these findings and support the hypothesis that patients with BPD show impaired responding to positive stimuli. Women with BPD might empathize with people in distress more easily than with people experiencing positive emotions, as they are more familiar with experiencing negative emotions themselves [14].

The finding that women with BPD scored lower on emotional empathy in both conditions might be due to a biased perception of ambiguous social situations. In our Cyberball game, co-players were represented by pictograms rather than real pictures, which prevented participants from reading facial expressions or emotions. There is evidence that patients with BPD anticipate social rejection or threat in such ambiguous social situations [13]. Additionally, women with BPD in our sample scored higher on rejection sensitivity which is also related to the expectancy of negative consequences [38]. Our results suggest that social exclusion is not the critical aspect of psychosocial stress that reduces emotional empathy in women with BPD.

### Cortisol and salivary alpha-amylase

Women with BPD and HC did not differ in basal cortisol or sAA levels. This matches findings of previous studies that also report no differences between patients with BPD and HC using single rather than continuous measurements of cortisol or sAA [15, 17]. As most previous studies report blunted cortisol reactivity to psychosocial stressors in patients with BPD [15, 17], we did not expect an increase in cortisol in women with BPD. We chose Cyberball as a social stressor, as it is known to not induce a physiological stress reaction [20, 21, 28, 29, 40]. In line with these studies, Cyberball did not result in an increase in cortisol patients with BPD and in HC. One possible explanation might be that cortisol’s main function is to mobilize energy and it is mostly triggered in stressful situations that demand active thinking, planning, or moving, which is not required in Cyberball [20]. Regarding sAA, we did not find any Cyberball effects, but increases over time, which corresponds with previous findings [2, 26]. The common elements of both Cyberball conditions or the experimental situation itself, such as social engagement with the researcher, may have activated an SNS response in participants.

### Cyberball as a psychosocial stressor

Based on results after psychosocial stress (TSST) [52], we had expected differences in empathy between Cyberball conditions. However, empathy did not differ between conditions and was only slightly reduced for positive emotions after exclusion. The TSST combines several stressful components besides social exclusion such as social evaluation, pressure to perform, ego-involvement as well as an increase in stress hormones and activation of the sympathetic nervous system. In contrast, Cyberball mainly induces exclusion and lacks these other stressful elements. Additionally, the Cyberball manipulation might have induced a weaker sense of exclusion than the TSST. In the TSST stress condition, participants are excluded by real people, while there are no people present in the control condition. Both Cyberball conditions, however, include no real human interactions and especially in the exclusion condition, participants only believed that they played with real people “a little”.

### Limitations

We tested effects of social exclusion in an artificial laboratory setting, which was one of the study’s main limitations. There was a high proportion of patients with comorbid diagnoses and intake of psychotropic medication, however, subgroup analyses yielded no effects on empathy. Patients with depression were excluded from the study, as HPA-axis function and cognition differ between patients with BPD with and without a depressive episode [51, 54]. As BPD is more frequently diagnosed in women, we tested only female participants and results are not generalizable to men.

### Conclusion and outlook

We conclude that women with BPD do not differ from healthy women in cognitive empathy and that this is not influenced by stress in general and social exclusion in particular. Emotional empathy, on the other hand, seems to be more sensitive to the effects of stress or ambiguous social situations in women with BPD. Specifically, emotional empathy seems to be reduced for positive emotions, and might further decline after social exclusion. Given these results, empathic reactions to emotional stimuli of different valences and to specific emotions should be further investigated. Future studies investigating the effects of social exclusion should use ecologically more valid Cyberball paradigms for example with virtual reality or a partial exclusion manipulation to increase believability. Next to social exclusion, other components of the TSST, such as social evaluation, one of the TSST’s most stressful components [20], should be addressed in further research.

## Supplementary Information

Below is the link to the electronic supplementary material.

Supplementary file1 (DOCX 43 KB)

## Data Availability

The authors confirm that the data supporting the findings of this study are available within the article and its supplementary materials. Further data are available from the corresponding author (KW) upon reasonable request.
