# Supplementary material for: Impact of social exclusion on empathy in women with borderline personality disorder
Source: Eur Arch Psychiatry Clin Neurosci. 2023 Jan 5;273(4):865–74. doi: 10.1007/s00406-022-01535-0 (PMC10238344; doi:10.1007/s00406-022-01535-0)
Supplement: Supplementary file 1 — Supplementary file1 (DOCX 43 KB) [file 406_2022_1535_MOESM1_ESM.docx]

**Supplementary Material**

**Methods**

Free cortisol was analyzed using an adapted homogenous time-resolved fluorescence resonance energy transfer (HTR-FRET)-based competitive immunoassay. Intra-assay coefficients of variation were below 8%, inter-assay coefficients of variation were below 10%. The limit of detection of free cortisol was 0.2 nM. All samples and standards were measured in duplicates. Alpha-amylase activity was determined using a modified protocol of a previously published direct alpha-amylase assay (Lorentz et al., Clin Chem Lab Med 1999; 37(11/12):1053–1062). Inter- and intra-assay coefficients of variation were both lower than 10 %. All samples and standards were measured in triplicates.

**Results**

**Demographics**

Of the BPD group 24 took one substance, 21 took two and five took three different substances. Patients reported taking the following substances: selective serotonin reuptake inhibitors (SSRI) *n* = 27, anti-psychotics *n* = 18, serotonin and noradrenaline reuptake inhibitors (SNRI) *n* = 14, dopamine and noradrenergic reuptake inhibitors (NDRI) *n* = 5, tricyclic antidepressants *n* = 3, anticonvulsants *n* = 2, noradrenergic and specific serotonergic antidepressants (NaSSa) *n* = 2, alpha/beta adrenergic blockers *n* = 1, methylphenidate *n* = 2.

In the BPD group the following comorbid diagnoses were determined: PTSD *n* = 25, eating disorder *n* = 18, alcohol abuse *n =* 5, drug abuse *n =* 5, agoraphobia with panic disorder *n* = 5, social phobia *n* = 4, panic disorder *n* = 3, ADHD *n* = 3, obsessive compulsive disorder *n* = 2, agoraphobia *n* = 1.

**Results Manipulation check**

Table A1

*Need Threat Questionnaire Results*

|  | **Group** | **Condition** | ***M*** | ***SD*** | **Statistics** |
| --- | --- | --- | --- | --- | --- |
| Need threat | BPD | Overincl. | 11.46 | 2.96 | group: *F*(1,192) = 86.52, *p* < .001, *η^2^* = *.*31 |
|  |  | Exclusion | 17.12 | 2.81 |  |
|  |  |  |  |  | condition: *F*(1,192) = 156.45, *p* < .001, *η^2^* = .45 |
|  | HC | Overincl. | 8.84 | 1.51 |  |
|  |  | Exclusion | 12.67 | 3.04 | group × condition: *F*(1,192) = 5.83, *p* = .017, *η^2^* = .03 |
| Ostra-cism intensity | BPD | Overincl. | 2.88 | 1.69 | group: *F*(1,192) = 22.37, *p* < .001, *η^2^ = .*10 |
|  |  | Exclusion | 8.59 | 1.84 |  |
|  |  |  |  |  | condition: *F*(1,192) = 496.96, *p* < .001, *η^2^* = .72 |
|  | HC | Overincl. | 2.19 | 0.70 |  |
|  |  | Exclusion | 7.04 | 2.05 | group × condition: *F*(1,192) = 3.31, *p* = .071, *η^2^* = .02 |
| Belief cover Story | BPD | Overincl. | 2.23 | 1.06 | group: *F*(1,189) = 6.33, *p* = .013, *η^2^* = *.*03 |
|  |  | Exclusion | 1.66 | 0.94 |  |
|  |  |  |  |  | condition: *F*(1,189) = 16.47, *p* < .001, *η^2^* = *.*08. |
|  | HC | Overincl. | 1.85 | 0.88 |  |
|  |  | Exclusion | 1.40 | 0.57 | group × condition: *F*(1,189) = 0.21, *p* = .648, *η^2^* = .00 |

*Note.* BPD = Borderline Personality Disorder, HC = Healthy controls, *M* = Mean, *SD* = Standard deviation

**Results Multidimensional Mood State Questionnaire (MDMQ)**

On the MDMQ subscale awake vs. tired we found a significant group effect (*F*(1,191) = 307.90, *p* < .001, *η^2^ =* .62). Women with BPD reported worse mood than HC regardless of condition. There was no significant condition effect or an interaction effect. There was also a significant within-subjects time effect (*F*(1,191) = 6.31, *p* = .014, *η^2^ =* .03). Mood decreased over time.

On the subscale awake vs. tired there was a group effect (*F*(1,191) = 132.60, *p* < .001, *η^2^* = .41), women with BPD reported feeling more tired than HC. There was also a condition effect (*F*(1,191) = 6.59, *p* = .011, *η^2^* = .03). Women in the exclusion condition reported feeling more tired than those in the overinclusion condition. We also found a time effect (*F*(1,191) = 9.98, *p* = .002, *η^2^ =* .05). Participants reported feeling more tired after the tasks than before.

On the subscale calm vs. nervous, there was a group effect (*F*(1,191) = 271.13, *p* < .001, *η^2^* = .59.) The BPD group reported feeling more nervous than the control group. We also found a condition effect (*F*(1,191) = 4.52, *p* = .035, *η^2^* = .02). Participants in the exclusion condition reported feeling more nervous than those in the overinclusion condition. For detailed information, see table A2.

Table A2

*Results of the Multidimensional Mood State Questionnaire (MDMQ)*

| **MDMQ**  **Scale** | **Group** | **Condition** | **Time** | ***M*** | ***SD*** | **Statistic** |
| --- | --- | --- | --- | --- | --- | --- |
| Good  vs. bad | BPD | Overincl. | Before | 12.86 | 3.02 | ***Between-subjects*** |
|  |  |  | After | 12.69 | 3.50 | group: *F*(1,191) = 307.90, *p* < .001, *η^2^ =* .62*** |
|  |  | Excl. | Before | 12.67 | 3.57 | condition: *F*(1,191) = 1.04, *p* = .308, *η^2^ =* .01 |
|  |  |  | After | 11.44 | 3.68 | group × condition: *F*(1,191) = 1.19, *p* = .277, *η^2^*= .01 |
|  | HC | Overincl. | Before | 18.48 | 1.24 | ***Within subjects*** |
|  |  |  | After | 18.35 | 1.62 | time: *F*(1,191) = 6.31, *p* = .014, *η^2^ =* .03* |
|  |  | Excl. | Before | 18.50 | 1.57 | time × group: *F*(1,191) = 3.01, *p* = .084, *η^2^ =*.02 |
|  |  |  | After | 18.38 | 1.55 | time × condition: *F*(1,191) = 2.57, *p* = .110, *η^2^ =.*01 |
|  |  |  |  |  |  | time × group × condition: *F*(1,191) = 2.62, *p* = .107, *η^2^ =.*01 |
| Awake vs. tired | BPD | Overincl. | Before | 11.98 | 3.80 | ***Between-subjects*** |
|  |  |  | After | 11.41 | 3.40 | group: *F*(1,191) = 132.60, *p* < .001, *η^2^* = .41*** |
|  |  | Excl. | Before | 10.77 | 3.63 | condition: *F*(1,191) = 6.59, *p* = .011, *η^2^* = .03* |
|  |  |  | After | 9.88 | 3.34 | group × condition: *F*(1,191) = 0.41, *p* = .523, *η ^2^*= .00 |
|  | HC | Overincl. | Before | 16.46 | 2.50 | ***Within subjects*** |
|  |  |  | After | 16.23 | 2.88 | time: *F*(1,191) = 9.98, *p* = .002, *η^2^ =* .05* |
|  |  | Excl. | Before | 15.68 | 2.71 | time × group: *F*(1,191) = 2.07, *p* = .152, *η^2^ =*.01 |
|  |  |  | After | 15.36 | 2.30 | time × condition: *F*(1,191) = 0.42, *p* = .516, *η^2^ =.*00 |
|  |  |  |  |  |  | time × group × condition: *F*(1,191) = 0.13, *p* = .715, *η^2^ =.*00 |
| Calm vs. nervous | BPD | Overincl. | Before | 11.41 | 3.03 | ***Between-subjects*** |
|  |  |  | After | 11.82 | 3.82 | group: *F*(1,191) = 271.13, *p* < .001, *η^2^* = .59*** |
|  |  | Excl. | Before | 10.23 | 3.69 | condition: *F*(1,191) = 4.52, *p* = .035, *η^2^* = .02* |
|  |  |  | After | 10.69 | 3.59 | group x condition: *F*(1,191) = 0.68, *p* = .411, *η^2^ =* .00 |
|  | HC | Overincl. | Before | 17.44 | 1.93 | ***Within subjects*** |
|  |  |  | After | 17.52 | 2.44 | time: *F*(1,191) = 1.64, *p* = .202, *η^2^ =* .01 |
|  |  | Excl. | Before | 17.00 | 2.36 | time × group: *F*(1,191) = 1.47, *p* = .226, *η^2^ =*.01 |
|  |  |  | After | 17.44 | 2.44 | time × condition: *F*(1,191) = 1.70, *p* = .247, *η^2^ =.*01 |
|  |  |  |  |  |  | time × group × condition: *F*(1,191) = 1.35, *p* = .247, *η^2^ =.*01 |

*Note.* BPD = Borderline Personality Disorder, HC = Healthy controls, *M* = Mean, *SD* = Standard deviation; *= *p* < .05, *** = *p* < .001

Table A3

*Multifaceted Empathy Test Results*

|  | **Group** | **Condition** | ***M*** | ***SD*** |
| --- | --- | --- | --- | --- |
| Cognitive empathy  (percent) | BPD | Overinclusion | 65.58 | 11.97 |
|  |  | Exclusion | 66.87 | 13.60 |
|  | HC | Overinclusion | 69.02 | 13.28 |
|  |  | Exclusion | 67.07 | 12.10 |
| Emotional empathy (mean) | BPD | Overinclusion | 5.59 | 1.26 |
|  |  | Exclusion | 5.11 | 1.33 |
|  | HC | Overinclusion | 5.83 | 1.21 |
|  |  | Exclusion | 5.80 | 1.18 |
| Emotional empathy positive emotions (mean) | BPD | Overinclusion | 5.09 | 1.65 |
|  |  | Exclusion | 4.35 | 1.58 |
|  | HC | Overinclusion | 5.93 | 1.27 |
|  |  | Exclusion | 6.08 | 1.31 |
| Emotional empathy negative emotions (mean) | BPD | Overinclusion | 5.97 | 1.40 |
|  |  | Exclusion | 5.70 | 1.55 |
|  | HC | Overinclusion | 5.75 | 1.50 |
|  |  | Exclusion | 5.59 | 1.35 |

*Note.* BPD = Borderline Personality Disorder, HC = Healthy controls, *M* = Mean, *SD* = Standard deviation
